# Pediatric Deaths during Measles Outbreak, Jerusalem, Israel, 2025–2026

**DOI:** 10.3201/eid3211.261397

**Published:** 2026-11

**Authors:** Zohar Mor, Hanna Shoob, Irinia Sokolov, Efrat Bucris, Chen Stein-Zamir, Ariel Tenenbaum, Orli Megged, Oren Gordon

**Affiliations:** Ministry of Health, Jerusalem, Israel (Z. Mor, H. Shoob, I. Sokolov); Ashkelon Academic College, Ashkelon, Israel (Z. Mor); Ministry of Health Virology Laboratory, Tel Hashomer, Israel (E. Bucris); Hebrew University of Jerusalem Faculty of Medicine, Jerusalem (C. Stein-Zamir, A. Tenenbaum, O. Megged, O. Gordon); Hadassah Ein Karem Medical Center, Jerusalem (A. Tenenbaum, O. Gordon); Shaare Zedek Medical Center, Jerusalem (O. Megged)

**Keywords:** measles, viruses, vaccine-preventable diseases, mortality, death, respiratory infections, fatality, MMR, pediatric, vaccine, Israel

## Abstract

Of 1,289 measles case-patients during April 2025–March 2026 in Jerusalem, Israel, 11 children died, a measles-specific mortality rate of 1.1 deaths/100,000 population. The measles-specific mortality rate among children <5 years of age was 7.7 deaths/100,000 population but 80.1 deaths/100,000 population in 6 neighborhoods populated by ultraorthodox Jews, a 47% measles-attributed death fraction.

In the United States, 2–3 deaths occur for every 1,000 reported measles cases (https://www.cdc.gov/surv-manual/php/table-of-contents/chapter-7-measles.html), a case-fatality rate (CFR) of 0.2%–0.3%. Overall, measles CFR is 0.3%–5.3% in low- and middle-income countries (*1*). The measles-specific mortality rate (MSMR) ranges from 0.04 deaths/100,000 population in Europe to 2.2 deaths/100,000 population in Africa (*2*). Immunosuppressed persons, malnourished persons, and children <5 years of age are at higher risk for death from measles (*3*).

The previous outbreak in Israel occurred during 2018–2019, during which 4,290 cases were reported (*4*). Of those cases, a total of 1,415 (33.0%) were in persons living in the city of Jerusalem (*5*,*6*), and 2 fatalities were reported (CFR 0.14%; MSMR 0.2 deaths/100,000 Jerusalem population). Jerusalem is the largest city in Israel, having a population of ≈1 million in 2025 (10.2% of the population of Israel), comprising ≈600,000 (62%) Jewish and ≈400,000 (38%) Arab persons (*7*). Of the Jewish population, ≈50.0% (≈300,000) were ultraorthodox religious Jews.

Coverage with the first measles, mumps, and rubella (MMR) and measles, mumps, rubella, and varicella (MMRV) vaccines among children 1–6 years of age in Jerusalem has decreased from 94.7% in 2019 to 78.3% in 2024 and is the lowest in Israel (https://datadashboard.health.gov.il/portal/dashboard/serviceQuality). Substantial geographic and ethnocultural differences exist across the city; coverage in April 2025 ranged from 99.4% among East Jerusalem Arab children to ≈55.0% in 6 designated neighborhoods (the 6 neighborhoods) in central Jerusalem, which has a population of ≈67,500, mostly ultraorthodox Jews.

We describe measles disease and death in Jerusalem during April 2025–March 2026. We also identify contributing factors to the unexpectedly elevated mortality rate.

## The Study

We determined measles diagnosis by using reverse transcription PCR of pharyngeal or urine specimens or by a combination of clinical symptoms and epidemiologic linkage to another measles case (https://www.gov.il/BlobFolder/policy/epi-92335924/he/files_regulation_epidemiology_epi-92335924.pdf). We sequenced samples by using Sanger or next-generation sequencing at the Ministry of Health’s Central Virology Laboratory. We conducted epidemiologic investigations on all cases to identify the source of exposure and define close contacts.

We calculated acute-phase measles mortality rates for all cases reported to surveillance teams during the 2025–2026 outbreak, expressing results as CFR and by MSMR because not all incident cases were reported. We calculated CFR by dividing measles-related deaths by all reported measles cases in a designated area, and calculated MSMR by dividing measles-related deaths by the total population in the designated area (Appendix).

During April 2025–March 2026, a total of 1,289 measles cases (129.2 cases/100,000 Jerusalem population) were reported in Jerusalem, comprising 36.1% of the total 3,574 cases reported in Israel (36.6 cases/100,000 Israel population). The median age of case-patients in Jerusalem was 3.1 (interquartile range [IQR] 1.5–5.4) years. Of all case-patients, 70.7% (912/1,289) were children <5 years of age. Median household size was 6 (IQR 5–8) persons, and 870 (95.4%) were unvaccinated for measles, whereas 42 (4.6%) received a vaccine single-dose. Most (885/1,289 [68.7%]) case-patients resided in the 6 neighborhoods, home to 6.8% of Jerusalem’s <5-year-old population, but 71.9% of <5-year-old case-patients in Jerusalem were reported in the 6 neighborhoods.

Of all 1,289 case-patients in Jerusalem, 11 died (64.7% of the 17 total deaths reported in Israel), yielding a CFR of 0.85% and an MSMR of 1.1 deaths/100,000 population (Table 1, Figure). The MSMR among children <5 years of age was 7.7 deaths/100,000 population in Jerusalem compared with 0.5 deaths/100,000 population in the rest of Israel. The MSMR among children <5 years of age in the 6 neighborhoods was 80.1 deaths/100,000 population, and the measles-attributed mortality fraction in that area compared with all-cause deaths during the previous year was 47%.

**Table 1:** Age-specific number of measles cases, number of measles-specific deaths, and case-fatality rates during measles outbreak, Jerusalem, Israel, April 2025–March 2026*

Characteristics	Jerusalem	Six neighborhoods in central Jerusalem
Total population, no.	997,870	67,530
No. reported cases of measles (measles cases/100,000 population)		
All reported measles cases	1,289 (129.2)	885 (1,310)
0–1 y	193 (825)	118 (5,254)
<5 y	912 (780)	656 (5,842)
5–9 y	272 (251)	193 (2,234)
<10 y	1,184 (525.4)	849 (4,273)
No. reported measles deaths (measles-specific deaths/100,000 population)		
All reported measles cases	11 (1.1)	11 (16.3)
0–1 y	1 (4.3)	1 (44.5)
<5 y	9 (7.7)	9 (80.1)
5–9 y	2 (1.8)	2 (23.1)
<10 y	11 (4.9)	11 (55.3)
Case-fatality rate, %		
All reported measles cases	0.85	1.2
0–1 y	0.5	0.85
<5 y	1.0	1.4
5–9 y	0.7	1.0
<10 y	0.9	1.3

*Age groups may overlap.

**Figure:**
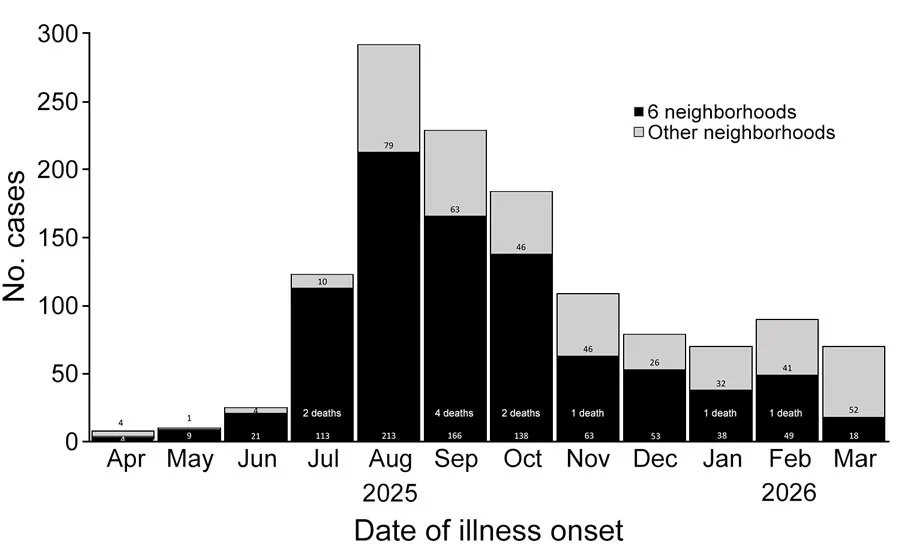
Number of reported measles cases and deaths, by month of illness onset, during measles outbreak, Jerusalem, Israel, April 2025–March 2026. Number of deaths per area and month are included.

The median age of children who died was 1.4 (IQR 1.1–2.5) years; 8 (72.7%) were boys and 3 (27.3%) were girls (Table 2). Ten of the children were otherwise healthy; 9 of those were <3 years of age, had not received any vaccinations, and were not registered in the mother-and-child health clinics. One 7-year-old child who died had known truncus arteriosus and was vaccinated with a single MMRV dose at 1 year of age, but did not receive the second dose.

**Table 2:** Demographic and other characteristics of children who died during measles outbreak, Jerusalem, Israel, April 2025–March 2026*

Case	Age, y/sex	No. measles vaccine doses	No. household members	Other vaccinations	No. ill household members	Days from first symptom to ED visit	Days from first symptom to death
1	2.4/M	0	10	None	2	9	27†
2	0.9/F	0	4	None	2	1	59†
3	1.3/M	0	7	None	1	8	38
4	1.3/M	0	12	None	1	15	15‡
5	1.4/M	0	14	None	1	19	19‡
6	1.9/M	0	6	None	2	7	7‡
7	2.5/F	0	10	None	1	4	15†
8	7.1/M	1	7	Partial	2	14	14‡
9	1.1/M	0	11	None	2	8	15
10	1.1/M	0	5	None	3	9	9‡
11	6.5/F	0	7	Partial	2	7	7‡

*ED, emergency department. †Patient received extracorporeal membrane oxygenation. ‡Patient dead on arrival at ED.

Analysis of the N450 region of the measles genome confirmed that 2 available samples from deceased children belonged to genotype B3. Those sequences matched the B3-named strain MVs/Quetta.PAK/44.20 (distinct sequence identifier 6418), which circulated in previous measles outbreaks in Europe (*8*,*9*).

The median household size of case-patients who died was 7 (IQR 6–11) persons, and 1–3 other family members reportedly had measles. Reverse transcription PCR–based diagnosis was confirmed in 9 (81.8%) children who died (Table 3), whereas 2 had clinical signs of measles and epidemiologic links. The median time from the first measles symptom (fever or rash) to emergency department (ED) visit was 8 (IQR 6–14) days, and death was reported within 15 (IQR 9–24) days. Six children were dead on arrival at the ED, and 5 children were hospitalized for a median of 21 (IQR 11–32) days; of those, 3 were treated with extracorporeal membrane oxygenation. Eight (72.7%) case-patients had radiologic findings indicative of pneumonia on chest radiograph at admission, and 6 case-patients had positive blood cultures

**Table 3:** Laboratory and clinical characteristics of children who died during measles outbreak, Jerusalem, Israel, April 2025–March 2026*

Case	Measles RT-PCR result	Fever	Rash	Cough	Conjunctivitis	Koplik spots	Pneumonia on chest x-ray	Blood culture findings	Respiratory aspirate or pleural fluid culture findings
1	+	Y	Y	N	N	N	Y	*Staphylococcus pneumoniae*	–
2	+	Y	Y		Y	Y	Y	–	–
3	+	Y	Y	Y	Y	Y	Y	–	–
4	NA	Y	Y	Y	N	N	Y	MSSA	–
5	NA	Y	Y	Y	N	N	Y	*S. pyogenes*	*S. pyogenes*
6	+	Y	Y	Y	N	N	N	MSSA and *Haemophilus influenzae* (non–B)	–
7	+	Y	Y	Y	Y	Y	Y	–	MSSA and *S. pyogenes*
8	+	Y	Y		N	N	N	–	–
9	+	Y	Y	Y	Y	Y	Y	–	MSSA
10	+	Y	Y	N	N	N	N	*S. pneumoniae*	–
11	+	Y	Y	N	N	N	Y	*S. pettenkoferi*	–
*MSSA, methicillin-susceptible *Staphylococcus aureus*; NA, not available; RT-PCR, reverse transcription PCR; +, positive; –, negative.									

One limitation of our study is the uncertainty about the actual number of measles cases in Jerusalem. A back-calculation based on the 11 observed measles death in Jerusalem and CFR estimates of 0.1%–0.3% in developed countries (https://www.ecdc.europa.eu/sites/default/files/documents/MEAS-AER-2024-Report.pdf; https://www.cdc.gov/surv-manual/php/table-of-contents/chapter-7-measles.html) suggest that the actual number of measles cases might have been substantially higher than the number reported in Jerusalem. Because of the possible underascertainment, we added an MSMR calculation, which is more stable and population-based. In addition, we only calculated acute measles deaths, not long-term mortality rates.

## Conclusions

The reported 2025–2026 measles disease rate in Jerusalem was 129.2 cases/100,000 population, which was 15 times higher than in the rest of Israel. CFR was 0.85% in Jerusalem and 0.26% in the rest of Israel, higher than the 0.1% reported in Europe (https://www.ecdc.europa.eu/sites/default/files/documents/MEAS-AER-2024-Report.pdf; https://measles-rubella-monthly.ecdc.europa.eu) and 0.2%–0.3% in the United States (https://www.cdc.gov/measles/data-research/index.html). The high MSMR among children <5 years of age also might reflect the overwhelming effect of the outbreak, which caused 80.1 deaths/100,000 children in the 6 neighborhoods in Jerusalem.

Prevention through vaccination is the mainstay in controlling measles outbreaks. Given the measles first-vaccine coverage of 55% in the 6 neighborhoods at the beginning of the outbreak, combined with MMR vaccine efficacy of 95% at 12 months (*3*), an estimated 47.7% of infants in each birth cohort were unprotected against measles. The young age of the unvaccinated measles case-patients, in addition to overcrowding and the presence of other sick household members, increased domestic measles transmission because of higher in-house viral inoculum and repeated exposures (*10*). In response, the Ministry of Health recommended MMR vaccine in Jerusalem at 6 months because maternal antibodies wane over time (*3*).

The late appearance in the ED of patients with fatal cases (i.e., 6/11 case-patients were dead upon arrival), might reflect avoidance of healthcare utilization in ultraorthodox communities because of distinct cultural, religious, and social considerations and insularity from government and secular media (*5*). A possible mistrust of government institutions might contribute to vaccine hesitancy, alongside frustration with isolation and coerced measures imposed during the COVID-19 pandemic and skepticism about MMR/MMRV vaccination.

Some parents reported they first consulted community mediators when the child’s condition deteriorated. Those mediators were known in the community for their medical backgrounds, having served as medical orderlies or midwives. The mediators used pulse oximeters to monitor the ill children, and some suggested vitamin A supplements or provided intravenous fluids before seeking conventional medical support, which might explain delayed referral to healthcare facilities. Underutilization of the healthcare system and low vaccination coverage underscore trust gaps between the ultraorthodox community and the public healthcare system, reflecting an intricate interplay of cultural, socioeconomic, and informational influences (*11*). A similar community response was demonstrated during COVID-19; prevalence of disease and death were much higher among ultraorthodox Jews in Israel compared with the overall population of Israel, but vaccine coverage remained low (*12*) among ultraorthodox Jewish persons in Israel and in other ultraorthodox Jewish communities elsewhere (*13*).

Additional factors contributing to the low level of trust and undervaccination might include a high spiritual locus of control. Persons with pronounced spiritual beliefs might be less inclined to vaccinate because of a belief in divine control over health outcomes, as found in a recent study in Israel (*14*).

Ultraorthodox communities comprise diverse subgroups, each guided by its own religious leadership and differing in their sources of influence, attitudes toward the community and external institutions, and relationships with wider society, all of which affect attitudes and behaviors toward vaccines and immunizations (*12*). Although rabbinical endorsement is essential to support a timely vaccination schedule, some indications suggest that the community is becoming less obedient to religious authority. The decrease in the influence of central religious authority, parallel with individual decision-making, is an opportunity to establish direct channels of communication between religious persons and healthcare providers to deliver direct health messages to ultraorthodox persons.

Secondary infection attributable to immune suppression and immune amnesia might have contributed to severe outcomes (*10*). Longer follow-up on children recovering from measles might identify additional cases of measles illness and death. That explanation is supported by the large number of case-patients who had bacteremia among all fatal cases (6 of the 11 children who died), whereas 2 were infected with vaccine-preventable pathogens.

In summary, we observed high MSMR and measles prevalence in Jerusalem during 2025–2026, mainly among unvaccinated children <5 years of age from ultraorthodox Jewish families. The 11 childhood deaths, including 6 in children who died before hospital arrival, represent a serious public health event in a city with advanced medical and vaccination services but where vaccination gaps exist. Although the previous 2018–2019 outbreak was successfully controlled, measles vaccine coverage has declined, partly because of changes in community behavior and the post–COVID-19 social climate. Outbreak vaccination efforts need to be followed by continuous maintenance of routine coverage. Culturally tailored interventions, developed in partnership with community leaders, are needed to strengthen trust in healthcare providers and vaccines, promote earlier healthcare-seeking among parents to enable timely recognition and medical assessment, reduce the risk for delayed diagnosis, and prevent clinical deterioration (*14*).

Appendix Additional information about pediatric deaths during measles outbreak, Jerusalem, Israel, 2025–2026.

## References

[1] Sbarra AN, Mosser JF, Jit M, Ferrari M, Ramshaw RE, O’Connor P, et al. Estimating national-level measles case-fatality ratios in low-income and middle-income countries: an updated systematic review and modelling study. Lancet Glob Health. 2023;11:e516–24. 10.1016/S2214-109X(23)00043-8 36925172 PMC10030458

[2] Antoni S, Ferrari MJ, Wimmer A, Nedelec Y, Steulet C, Gacic-Dobo M, et al. Progress towards measles elimination—worldwide, 2000–2024. Weekly epidemiological record. 2025 Nov 28 [cited 2026 Jul 10]. https://iris.who.int/server/api/core/bitstreams/8c65dd54-ee98-4438-938c-e7114851f870/content

[3] Do LAH, Mulholland K. Measles 2025. N Engl J Med. 2025;393:2447–58. 10.1056/NEJMra2504516 40561553

[4] Anis E, Haas EJ, Indenbaum V, Singer SR, Warshavsky B, Rishpon S, et al. A prolonged, nationwide measles outbreak despite very high vaccination coverage in Israel, 2018-19. J Infect. 2021;83:678–85. 10.1016/j.jinf.2021.09.025 34626701

[5] Stein-Zamir C, Abramson N, Shoob H. Large measles outbreak in orthodox Jewish communities—Jerusalem District, Israel, 2018–2019. MMWR Morb Mortal Wkly Rep. 2020;69:562–3. 10.15585/mmwr.mm6918a3 32379730 PMC7737952

[6] Stein-Zamir C, Shoob H, Abramson D. Measles clinical presentation, hospitalization and vaccination status among children in a community-wide outbreak. Vaccine. 2023;41:2764–8. 10.1016/j.vaccine.2023.03.043 37002179

[7] Jerusalem Institute for Policy Research. Jerusalem statistical yearbook 2026. 2026 [cited 2026 Jun 27]. https://jerusaleminstitute.org.il/en/yearbook/#/4372/31934

[8] Bucris E, Zuckerman NS, Levin T, Kanaaneh Y, Erster O, Indenbaum V, et al.; National Measles Working Group. National Measles Working Group. Genomic and epidemiologic insights into ongoing measles outbreak, Israel, 2025–2026. Emerg Infect Dis. 2026;32:1678–82. 10.3201/eid3209.260625 42497067 PMC13555727

[9] Cassini A, Cobuccio L, Glampedakis E, Cherpillod P, Crisinel PA, Pérez-Rodríguez FJ, et al. Adapting response to a measles outbreak in a context of high vaccination and breakthrough cases: an example from Vaud, Switzerland, January to March 2024. Euro Surveill. 2024;29:2400275. 10.2807/1560-7917.ES.2024.29.22.2400275 38818746 PMC11141130

[10] Jacobson A, Spitzer S, Gorelik Y, Edelstein M. Barriers and enablers to vaccination in the ultra-orthodox Jewish population: a systematic review. Front Public Health. 2023;11:1244368. 10.3389/fpubh.2023.1244368 37900036 PMC10602685

[11] Spitz A, Siman-Tov M, Shapiro E. The complex issue of trust of Israel’s Jewish religious community in health care and religious institutions involved in addressing the COVID-19 pandemic. J Relig Health. 2026;65:573–93. 10.1007/s10943-025-02436-x 40924291

[12] Berger Lipsky T, Gabbay E. Sociocultural and religious perspectives toward the COVID-19 pandemic in the Haredi Jewish community. J Relig Health. 2023;62:389–407. 10.1007/s10943-022-01667-6 36115889 PMC9483331

[13] Gendler Y, Ofri L, Videl H. Understanding factors contributing to vaccination underutilization among Jewish ultra-orthodox communities in Israel: a cross-sectional study. Vaccine. 2025;47:126711. 10.1016/j.vaccine.2025.126711 39793538

[14] Kasstan B. “If a rabbi did say ‘you have to vaccinate,’ we wouldn’t”: Unveiling the secular logics of religious exemption and opposition to vaccination. Soc Sci Med. 2021;280:114052. 10.1016/j.socscimed.2021.114052 34051560 PMC9755973

