# Supplementary material for: Pediatric Deaths during Measles Outbreak, Jerusalem, Israel, 2025–2026
Source: Emerg Infect Dis. 2026 Nov;32(11):1865–9. doi: 10.3201/eid3211.261397 (PMC13715882; doi:10.3201/eid3211.261397)
Supplement: Supplementary file 1 [file 26-1397-Techapp-s1.pdf]

Article DOI: https://doi.org/10.3201/eid3211.261397

EID cannot ensure accessibility for supplementary materials supplied by authors. Readers who have difficulty accessing supplementary content should contact the authors for assistance.

Pediatric Deaths during Measles Outbreak, Jerusalem, Israel, 2025–2026

Appendix

During April 2024–March 2025, when no measles cases were reported, 10 fatalities of all causes were reported among 11,200 children younger than 5 years of age who lived in the 6 neighborhoods in central Jerusalem populated with ultraorthodox Jews.

During April 2025–March 2026, a total of 656 measles cases were reported in children younger than 5 years of age who lived in those 6 neighborhoods in central Jerusalem, and 19 fatalities of all causes.

Attributable fraction =

$$\frac{Death\ between\ April\ 2025\ to\ March\ 2026 - Death\ between\ April\ 2024\ and\ March\ 2025}{Death\ between\ April\ 2025\ to\ March\ 2026} = \frac{19/11{,}200 - 10/11{,}200}{19/11{,}200} = 0.47$$
